# Transmitted drug resistance and transmission clusters among HIV-1 treatment-naïve patients in Guangdong, China: a cross-sectional study

**DOI:** 10.1186/s12985-021-01653-6

**Published:** 2021-09-06

**Authors:** Yun Lan, Linghua Li, Xiang He, Fengyu Hu, Xizi Deng, Weiping Cai, Junbin Li, Xuemei Ling, Qinghong Fan, Xiaoli Cai, Liya Li, Feng Li, Xiaoping Tang

**Affiliations:** 1 Guangzhou Eighth People’s Hospital, Guangzhou Medical University, 627 Dongfeng East Road, Yuexiu District, Guangzhou, 510060 China; 2 Guangdong Center for Diagnosis and Treatment of AIDS, 627 Dongfeng East Road, Yuexiu District, Guangzhou, 510060 China; 3 Guangdong Provincial Institute of Public Health, Guangdong Provincial Center for Disease Control and Prevention, 160 Qunxian Road, Panyu District, Guangzhou, 511430 China

**Keywords:** HIV-1, Transmitted drug resistance, Transmission cluster, Guangdong

## Abstract

**Background:**

Transmitted drug resistance (TDR) that affects the effectiveness of the first-line antiretroviral therapy (ART) regimen is becoming prevalent worldwide. However, its prevalence and transmission among HIV-1 treatment-naïve patients in Guangdong, China are rarely reported. We aimed to comprehensively analyze the prevalence of TDR and the transmission clusters of HIV-1 infected persons before ART in Guangdong.

**Methods:**

The HIV-1 treatment-naïve patients were recruited between January 2018 and December 2018. The HIV-1 pol region was amplified by reverse transcriptional PCR and sequenced by sanger sequencing. Genotypes, surveillance drug resistance mutations (SDRMs) and TDR were analyzed. Genetic transmission clusters among patients were identified by pairwise Tamura-Nei 93 genetic distance, with a threshold of 0.015.

**Results:**

A total of 2368 (97.17%) HIV-1 *pol* sequences were successfully amplified and sequenced from the enrolled 2437 patients. CRF07_BC (35.90%, 850/2368), CRF01_AE (35.56%, 842/2368) and CRF55_01B (10.30%, 244/2368) were the main HIV-1 genotypes circulating in Guangdong. Twenty-one SDRMs were identified among fifty-two drug-resistant sequences. The overall prevalence of TDR was 2.20% (52/2368). Among the 2368 patients who underwent sequencing, 8 (0.34%) had TDR to protease inhibitors (PIs), 22 (0.93%) to nucleoside reverse transcriptase inhibitors (NRTIs), and 23 (0.97%) to non-nucleoside reverse transcriptase inhibitors (NNRTIs). Two (0.08%) sequences showed dual-class resistance to both NRTIs and NNRTIs, and no sequences showed triple-class resistance. A total of 1066 (45.02%) sequences were segregated into 194 clusters, ranging from 2 to 414 sequences. In total, 15 (28.85%) of patients with TDR were included in 9 clusters; one cluster contained two TDR sequences with the K103N mutation was observed.

**Conclusions:**

There is high HIV-1 genetic heterogeneity among patients in Guangdong. Although the overall prevalence of TDR is low, it is still necessary to remain vigilant regarding some important SDRMs.

## Background

Guangdong is one of the areas in China most heavily affected by HIV-1. By the end of October 2019, Guangdong reported the fourth highest number of HIV cases (66,558) in China [1]. National wide antiretroviral therapy (ART) has substantially curbed rampant HIV transmission [2] and has significantly reduced the HIV infection associated mortality and morbidity [3, 4]. However, emerging HIV drug resistant variants due to the long-term ART selection post a threat to HIV prevention and control [5].

Transmitted drug resistance (TDR) of HIV is prevalent but varies worldwide. For example, the prevalence of TDR of HIV has been reported to be 4.1% in south/southeast Asia and 6.0% in sub-Saharan Africa [6] 14% in southwestern Siberia [7], 7.8% in Greece [8], 8.0% in Brighton [9], and 13.1% in Portugal [10]. In 2015, a nationwide cross-sectional survey revealed that the overall prevalence of TDR was 3.6% in China [11]. More recently, the TDR rate of many cities in China has increased 4.5% in Beijing [12], 7.21% in Guangxi [13], 11.1% in Zhejiang [14], and 7.8% in Tianjin [15].

Molecular transmission clusters can be identified by molecular phylogeny based on evolutionary theory and sequence analysis [16, 17]. The analysis of transmission clusters has been widely used to study HIV-1 transmission kinetics and develop real-time precision interventions [18, 19]. International guidelines recommend that newly diagnosed HIV patients should be tested for ART drug resistance for potential TDR and for antiviral drug selection [16, 17]. Given that first-line ART drugs has been used in Guangdong for thirty years, it is essential to investigate the prevalence and transmission of TDR among HIV-1-infected adults in Guangdong. Here, we performed a large cohort cross-sectional study in ART-naïve HIV-1-infected individuals in Guangdong.

## Methods

### Study population

Between January 2018 and December 2018, 2368 HIV-1 patients were enrolled in this study based on the following criteria (1) adult residents being over 16 years old and living in Guangdong Province; (2) diagnosed with HIV infection within 3–6 months and never received ART; and (3) not infected via mother-to-infant transmission. The epidemiological data of the patients (includingage, sex, marital status, education level, ethnicity, route of infection, and CD4^+^ T cell count) were acquired from the China Information System for Disease Control and Prevention.

### HIV-1 RNA extraction and *pol* gene amplification

The blood sample mixed with the anticoagulant ethylene diamine tetraacetic acid (EDTA) was centrifuged at 3000 rpm for 5 min to collect plasma. Viral RNA was extracted from the plasma using the QIAamp Viral RNA Mini Kit (Qiagen, Germany) following the manufacturer’s instructions. The extracted RNA was transcribed and nest amplified using the PrimeScript One Step RT-PCR Kit (Takara, China) and PrimeSTAR HS DNA Polymerase (Takara, China). The PCR products were analysed using agarose gel electrophoresis, and the positive products (approximately 1300 bp in the HIV-1 pol gene corresponding to HXB2 2147–3462 nt, encoding the protease and the first 299 residues of reverse transcriptase) were sent for ABI3730 sequencing in a commercial company (Tianyi Huiyuan, China). The sequences obtained were assembled and cleaned with Sequencher software.

### Genotype determination and analysis

Sequences were aligned, adjusted manually and merged with HIV-1 subtyping references downloaded from the Los Alamos HIV Sequence Database via Bioedit software. To determine the HIV-1 genotypes, sequences were assessed with the Context-based Modeling for Expeditious Typing (COMET) genotyping tool, developed by Daniel Struck [20] and the REGA HIV-1 Subtyping Tool Version 3.0, developed by Tulio de Oliveira [21]. The ML phylogenetic tree was used for confirmation. The phylogenetic tree was constructed using the maximum likelihood method with the GTR substitution model with the PhyML program 3.0 [22], and the branch support value was estimated using the approximate likelihood ratio test (aLRT) [23].

### TDR and drug resistance mutation analysis

TDR was defined as the presence of surveillance drug resistance mutation (SDRM) [10]. The Stanford Calibrated Population Resistance (CPR) tool 8.0 (last updated on 1st July 2019) was used to identify SDRMs according to the WHO 2009 surveillance list [21]. The Stanford HIVdb Program 8.9 (last updated on 7th Oct. 2019) was used to infer resistance to antiretroviral drugs, including protease inhibitors (PIs), nucleoside reverse transcriptase inhibitors (NRTIs) and non-nucleoside reverse transcriptase inhibitors (NNRTIs) [24]. Sequences with low-level, intermediate-level, or high-level resistance were defined as drug resistant.

### Transmission cluster construction

The HyPhy program 2.2.4 was used to calculate the pairwise Tamura-Nei 93 (TN93) genetic distance for the aligned sequences [25]. The network visualisation program Cytoscape 3.2.1 was used to analyse sequences with a threshold genetic distance of 0.015 and to visualize the transmission network as nodes (sequences), edges (links) and clusters (groups of linked sequences) [26]. This genetic distance threshold has been validated to identify partners with epidemiological links [27] and has been widely used [28, 29].

### Statistical analysis

All statistical analyses were performed using IBM SPSS program version 25.0. Qualitative statistics are described using the frequency. Quantitative statistics are described using the median (IQR). Univariate and multivariate logistic regression analyses were performed to identify potential risk factors. A *P*-value < 0.05 was considered statistically significant. Variables with a *P*-value < 0.05 in the univariate logistic regression analysis were included in the multivariate logistic regression analysis. Odds ratios (*ORs*) and adjusted odds ratios (a*ORs*) with their 95% confidence intervals (95% *CIs*) are reported.

## Results

### Demographic and clinical characteristics of the subjects

A total of 2368 (97.17%) HIV-1 *pol* sequences were successfully amplified and sequenced from the enrolled 2,437 participants whose age ranged from 16 to 90 years, with a median age of 36 years. In total, 86.53% (2,049/2,368) of the subjects were male. The most common infection route was men who have sex with men (MSM 46.75%, 1107/2368), followed by heterosexuals (HETs 42.40%, 1004/2368) and intravenous drug users (IDUs3.38%, 80/2,368. Approximately half of the participants were unmarried (46.28%, 1096/2368), and 36.95% were married or cohabiting (875/2368). The educational status of the subjects was mainly junior high school (34.76%, 823/2368). The median (range) CD4^+^ T cell count was 247 (1–1425) cells/mm^3^, and 37.80% (895/2368) of the subjects exhibited a CD4^+^ T cell count of < 200 cells/mm^3^ (Table 1).

**Table 1:** Demographic characteristics and factors associated with drug resistance

Variable	Number	TDR, N (%)	Crude OR (95% CI)	*P*-value	Adjusted OR(95% CI)	*P*-value
Total	2368	52 (2.2)				
**Age (years)**						
< 35	1097	25 (2.3)	1.000			
35–49	737	15 (2.0)	0.891 (0.466–1.701)	0.726		
≥ 50	534	12 (2.2)	0.986 (0.491–1.978)	0.968		
**Marital status**						
Unmarried	1096	22 (2.0)	1.000			
Married	875	16 (1.8)	0.909 (0.475–1.742)	0.774		
Divorce or widow	289	10 (3.5)	1.750 (0.819–3.738)	0.149		
Unknown	108	4 (3.7)	1.878 (0.635–5.552)	0.255		
**Education**						
Primary and below	330	4 (1.2)	1.000			
Junior high school	823	17 (2.1)	1.719 (0.574–5.147)	0.333		
Senior high school	551	12 (2.2)	1.814 (0.580–5.673)	0.306		
College and Above	586	16 (2.7)	2.288 (0.758–6.901)	0.142		
Unknown	78	3 (3.8)	3.260 (0.715–14.873)	0.127		
**Ethnicity**						
Han	2202	48 (2.2)	1.000			
Ethnic minorities	88	1 (1.1)	0.516 (0.070–3.780)	0.515		
Unknown	78	3 (3.8)	1.795 (0.547–5.894)	0.335		
**Transmission route**						
HET	1004	19 (1.9)	1.000			
MSM	1107	26 (2.3)	1.247 (0.686–2.267)	0.469		
IDU	80	1 (1.3)	0.656 (0.087–4.996)	0.683		
Other	177	6 (3.4)	1.819 (0.716–4.620)	0.208		
**CD4**^**+**^** T cell count(cells/mm3)**						
< 200	895	15 (1.7)	1.000		1.000	
200–499	1220	23 (1.9)	1.127 (0.585–2.173)	0.721		
≥ 500	253	14 (5.5)	3.437 (1.636–7.219)	0.001	4.062 (1.904–8.668)	< 0.001
**Genotype**						
CRF01_AE	842	24 (2.9)	1.000		1.000	
CRF07_BC	850	10 (1.2)	0.406 (0.193–0.854)	0.017	0.360 (0.170–0.764)	0.008
CRF08_BC	66	2 (3.0)	1.065 (0.246–4.608)	0.933		
CRF55_01B	244	5 (2.0)	0.713 (0.269–1.889)	0.496		
CRF59_01B	53	1 (1.9)	0.655 (0.087–4.941)	0.682		
Subtype B	70	3 (4.3)	1.526 (0.448–5.199)	0.499		
Other^a^	243	7 (2.9)	1.011 (0.430–2.375)	0.980		

### Distribution of HIV-1 genotypes

**Fig. 1:**
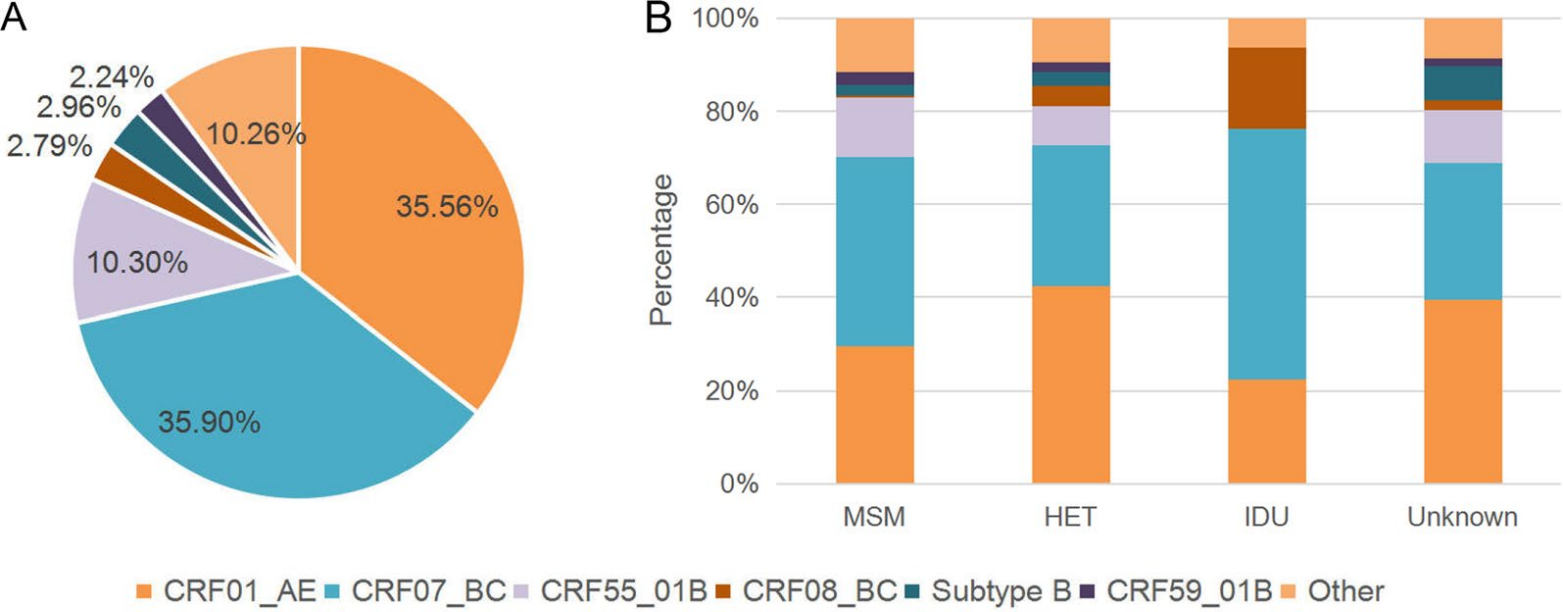
Genotypic analysis based on the sequences of the human immunodeficiency virus 1 *pol* gene. **A** Distribution of genotypes according to the HIV-1 *pol* gene. **B** Distribution of HIV-1 genotypes in each risk group. Other genotypes include subtype C subtype G, CRF02_AG, CRF12_BF, and some recombinant forms

The main HIV-1 genotypes circulating in Guangdong were found to be CRF07_BC (35.90%, 850/2368), CRF01_AE (35.56%, 842/2368) and CRF55_01B (10.30%, 244/2368), accounting for 81.76% of total infections. HIV-1 subtype B (2.96%, 70/2368), CRF08_BC (2.79%, 66/2368) and CRF59_01B (2.24%, 53/2368) were less frequently observed. HIV-1 Subtype C (0.46%, 11/2368), subtype G (0.13%, 3/2368), CRF02_AG (0.1%, 3/2368) and CRF12_BF (0.04%, 1/2368) were classified as minor in this study. In addition, 225 recombinant strains were observed (REGA tool ‘Recombination’, ‘Recombination-like’, ‘potential-Recombination’, or ‘check the report’; and COMET tool ‘unassigned’ and not clustered with any reference sequences by the phylogenetic tree). Minor HIV-1 genotypes and recombinant strains were classified as ‘other’ genotypes (Fig. 1A).

The distribution of HIV-1 genotypes varied among different risk groups (Fig. 1B). CRF07_BC (40.65%, 450/1107), CRF01_AE (29.63%, 328/1107) and CRF55_01B (12.74%, 141/1107) were the dominant genotypes circulating among MSM, and CRF08_BC (0.36%, 4/1107) was rarely detected in this risk group. CRF01_AE (42.43%, 426/1004), CRF07_BC (30.28%, 305/1004) and CRF55_01B (8.27%, 83/1004) were the main genotypes circulating among HETs. CRF07_BC accounted for more than half of the genotypes circulating among IDUs (53.75%, 43/80), followed by CRF01_AE (22.50%, 18/80) and CF08_BC (17.50%, 14/80).

### HIV drug resistance mutations (SDRMs)

**Fig. 2:**
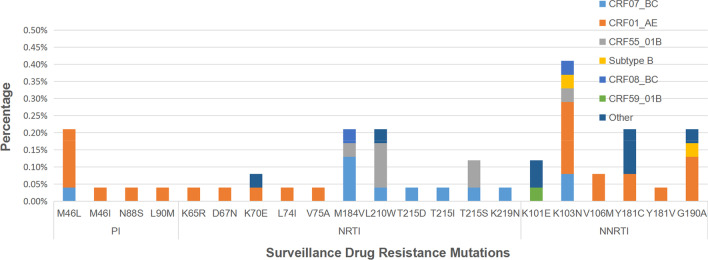
Distribution of surveillance drug resistance mutations among HAART-naïve HIV-1-infected individuals from Guangdong China

Twenty-one SDRMs were identified among fifty-two drug-resistant strains by the CPR program. M46L (0.17%, 4/2368) was the most prevalent mutation in the protease region. K103N (0.42%, 10/2368), Y181C (0.21%, 5/2368), and G190A (0.21%, 5/2368) were the most common NRTI-associated mutations, and M184V (0.21%, 5/2368), L210W (0.21%, 5/2368), and T215S (0.13%, 3/2368) were the most common NNRTI-associated mutations (Fig. 2). Patients infected with the CRF01_AE (0.29%) strain were most likely to acquire a PI-associated SDRM, followed by those infected with the CRF07_BC strain (0.04%). Patients infected with the CRF07_BC strain were most likely to acquire an NRTI-associated SDRM, followed by those infected with the CRF01_AE strain and CRF55_01B strain. Patients infected with the CRF01_AE strain were most likely to acquire an NNRTI-associated SDRM, followed by those infected with the CRF07_BC and subtype B strains (Fig. 2).

### HIV TDR and its associated factors

The clinical impact of these mutations was assessed with the Stanford HIVdb tool. In total 2.20% (52/2368) of patients had TDR (Table 2). Among them, 8 (0.34%) had TDR to PIs, 22 (0.93%) to NRTIs, and 23 (0.97%) to NNRTIs (Table 2). Two (0.08%) strains showed dual-class resistance to NRTIs and NNRTIs, and no strains showed triple-class resistance. For NNRTIs, the most frequent TDR drugs were EFV and NVP (all 1.01%, 24/2368). For NRTIs, the most frequent TDR drug was D4T (0.63%, 15/2368), followed by AZT (0.46%, 11/2368). All seven patients with TDR to PIs were resistant to NFV.

**Table 2:** Transmission drug resistance among ART naïve HIV-1 infections from Guangdong China

Subtypes	Number	Number of TDR	Prevalence (%)	Prevalence (%)		
				PI	NRTI	NNRTI
CRF07_BC	850	10	1.18	0.12 (1/850)	0.82 (7/850)	0.24 (2/850)
CRF01_AE	842	24	2.85	0.71 (6/842)	0.95 (8/842)	1.31 (11/842)
CRF55_01B	244	5	2.05	0	1.64 (4/244)	0.41 (1/244)
Subtype B	70	3	4.29	1.43 (1/70)	0	2.86 (2/70)
CRF08_BC	66	2	3.03	0	1.52 (1/66)	1.52 (1/66)
CRF59_01B	53	1	1.89	0	(0/53)	1.89 (1/53)
Other	243	7	2.88	0	0.82 (2/243)	2.06 (5/243)
* Subtype C*	11	2	18.18	0	0	18.18 (2/11)
* Subtype G*	3	0	0	0	0	0
* CRF02_AG*	3	0	0	0	0	0
* CRF12_BF*	1	0	0	0	0	0
Recombinant strain	225	5	2.22	(0/225)	0.89 (2/225)	1.33 (3/225)
Total	2368	52	2.20	0.34 (8/2368)	0.93 (22/2368)	0.97 (23/2368)

TDR, transmission drug resistance; PI, protease inhibitor; NRTI, nucleoside reverse transcriptase inhibitor; NNRTI, non-nucleoside reverse transcriptase inhibitor

Risk factors associated with HIV TDR are listed in Table 1. In the univariate logistic regression analysis, two factors were significantly associated with HIV TDR. The *OR* for patients whose CD4^+^ T cell count was above 500 cells/mm^3^ versus patients whose CD4^+^ T cell count was below 200 cells/mm^3^ was 3.437 (95% *CI *1.636–7.219) and that for patients infected with the CRF07_BC strain versus patients infected with the CRF01_AE strain was 0.406 (95% *CI *0.193–0.854). The multivariate logistic regression model showed that a CD4^+^ T cell count above 500 cells/mm^3^ and CRF07_BC were important risk factors, with a*OR*s of 4.062 (95% *CI* 1.904–8.668) and 0.360 (95% *CI* 0.170–0.764), respectively.

### Genetic transmission cluster analysis

**Fig. 3:**
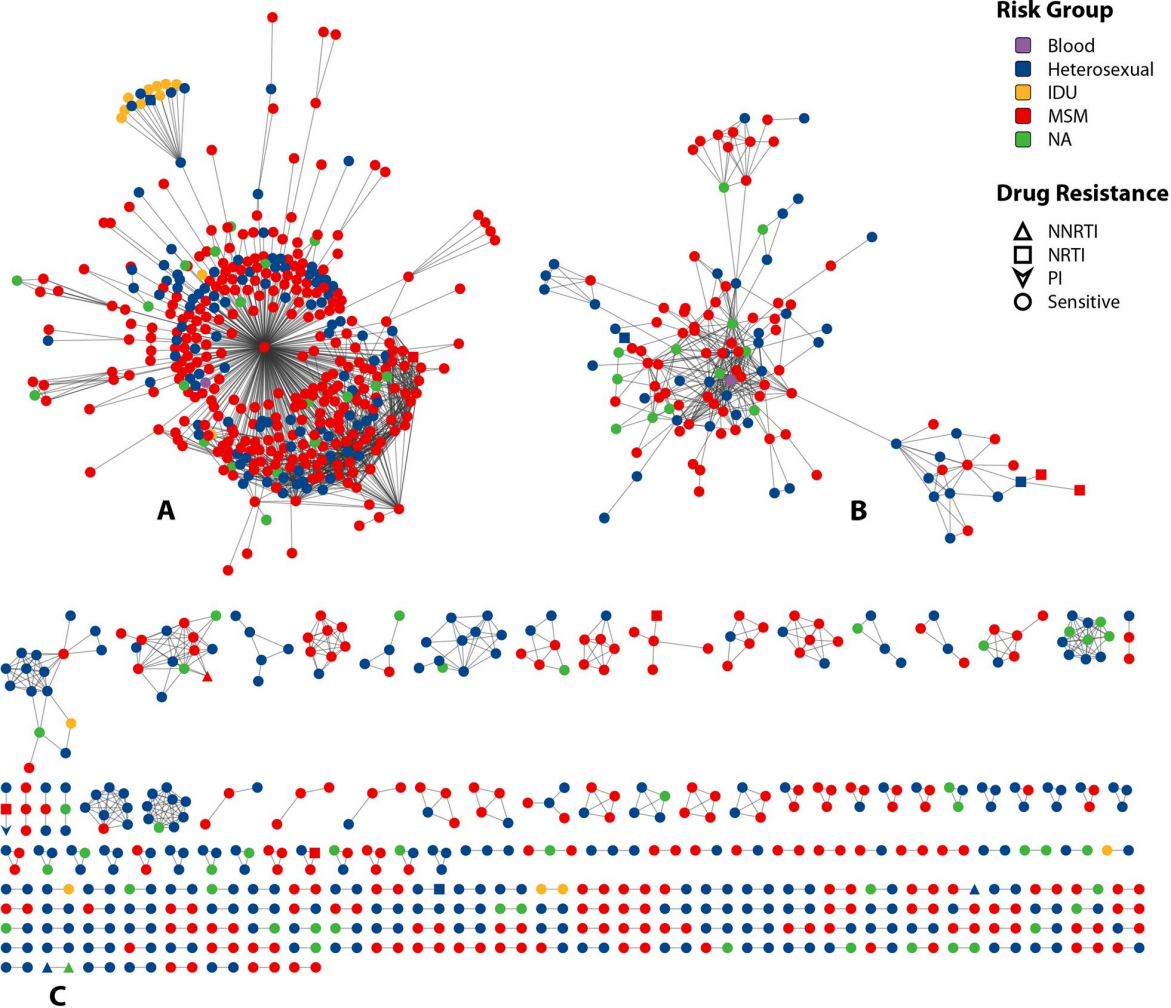
Risk factor- and drug resistance-associated genetic transmission networks. **A** The largest cluster included 414 CRF07_BC sequences. **B** The second largest cluster included 1124 CRF55_01B sequences. **C** The CRF01_AE cluster with the surveillance drug resistance mutation K103N

All 2368 sequences were used to construct the genetic transmission network, of which 1066 (45.02%) were segregated into 194 clusters with a genetic distance threshold of 1.5%, ranging from 2 to 414 sequences (Fig. 3). A total of 93.30% (181/194) of clusters had a size ≤ 5 and 6.70% (13/194) of clusters had a size > 5. The largest cluster A was the CRF07_BC cluster with 414 sequences, followed by the CRF55_01B cluster B with 124 sequences (Fig. 3). A total of 50.86% (563/1107) of sequences from MSM were included in the networks and dispersed among 53.09% (103/194) of the transmission networks, and 40.64% (408/1004) of sequences from HETs were included in the networks and dispersed among 69.59% (135/194) of the transmission networks. We also observed that 28.85% (15/52) of patients with TDR were included in 9 clusters, and an analysis of shared mutations revealed that cluster C contained two TDR sequences with the K103N mutation (Fig. 3). The proportion of patients with TDR entering the network was lower than that of those without TDR, and the difference was statistically significant (χ^2^ = 5.617, p = 0.023 < 0.05). These individuals with TDR included 10 patients with resistance to NRTIs, 4 patients with resistance to NNRTIs, and 1 patient with resistance to PIs.

Patients were divided according to whether they entered the transmission network, and the risk factors listed in Table 3 were examined. The multivariate logistic regression model showed that infection through intravenous drug use, a CD4^+^ T cell count between 200 and 499 cells/mm^3^, and CRF07_BC or CRF55_01B were important factors, with a*OR*s of 0.266 (95% *CI* 0.144–0.493), 1.339 (1.095–1.636), 3.435 (2.789–4.232) and 2.498 (95% *CI* 1.850–3.372), respectively (Table 3).

**Table 3:** Factors associated with transmission within clusters

Variable	Number	Persons in TC, N (%)	Crude OR (95% CI)	*P*-value	Adjusted OR(95% CI)	*P*-value
Total	2368					
**Age (years)**						
< 35	1097	527 (48.0)	1.000		1.000	
35–49	737	292 (39.6)	0.710 (0.587–0.858)	< 0.001	0.857(0.661–1.111)	0.244
≥ 50	534	247 (46.3)	0.931 (0.757–1.145)	0.498		
**Marital status**						
Unmarried	1096	520 (47.4)	1.000		1.000	
Married	875	382 (43.7)	0.858 (0.718–1.026)	0.940		
Divorce or widow	289	118 (40.8)	0.764 (0.588–0.994)	0.045	0.811(0.569–1.155)	0.246
Unknown	108	46 (42.6)	0.822 (0.551–1.225)	0.336		
**Education**						
Primary and below	330	134 (40.6)	1.000		1.000	
Junior high school	823	355 (43.1)	1.110 (0.856–1.438)	0.432		
Senior high school	551	259 (47.0)	1.297 (0.984–1.710)	0.065		
College and above	586	286 (48.8)	1.394 (1.061–1.832)	0.017	0.997(0.697–1.424)	0.986
Unknown	78	32 (41.0)	1.018 (0.616–1.681)	0.946		
**Ethnicity**						
Han	2202	1000 (45.4)	1.000			
Ethnic minorities	88	34 (38.6)	0.757 (0.489–1.172)	0.212		
Unknown	78	32 (41.0)	0.836 (0.528–1.323)	0.445		
**Route of infection**						
Heterosexual intercourse	1004	408 (40.6)	1.000		1.000	
Homosexual intercourse	1107	563 (50.9)	1.512 (1.272–1.796)	< 0.001	1.451(1.156–1.821)	0.001
Intravenous drug use	80	16 (20.0)	0.365 (0.208–0.641)	< 0.001	0.266(0.144–0.493)	< 0.001
Others	177	79 (44.6)	1.178 (0.853–1.625)	0.320		
**CD4**^**+**^** T cell count(cells/mm3)**						
< 200	895	358 (40.0)	1.000		1.000	
200–499	1220	598 (49.0)	1.442 (1.211–1.717)	< 0.001	1.339 (1.095–1.636)	0.004
≥ 500	253	110 (43.5)	1.154 (0.870–1.530)	0.320		
**Genotype**						
CRF01_AE	842	305 (36.2)	1.000		1.000	
CRF07_BC	850	560 (65.9)	3.400 (2.785–4.151)	< 0.001	3.435 (2.789–4.232)	< 0.001
CRF08_BC	66	12 (18.2)	0.391 (0.206–0.743)	0.004	0.488 (0.252–0.947)	0.034
CRF55_01B	244	145 (59.4)	2.579 (1.926–3.452)	< 0.001	2.498 (1.850–3.372)	< 0.001
CRF59_01B	53	22 (41.5)	1.249 (0.711–2.197)	0.439		
Subtype B	70	22 (31.4)	0.807 (0.478–1.363)	0.422		
Other	243	0 (0.0)	–			
**Drug resistance**						
Yes	52	15 (28.8)	1.000		1.000	
No	2316	1051 (45.4)	2.049 (1.119–3.755)	0.020	1.709 (0.884–3.302)	0.111

TC, transmission cluster; OR, odd ration; CI, confidence interval; MSM, men who have sex with men; HET, heterosexual; IDU, intravenous drug use; CRF, circulating recombinant form

## Discussion

In this study, we investigated the genetic characteristics and prevalence of TDR among ART-naïve HIV-1-infected individuals newly diagnosed in Guangdong, China, in 2018. The major epidemic HIV-1 genotypes detected in Guangdong were CRF07_BC (35.90%), CRF01_AE (35.56%), and CRF55_01B (10.30%). The distribution of HIV-1 genotypes in Guangdong has changed over the last three decades. Before 2000, subtype C (46.2%) and subtype B (30.7%) were the major prevalent strains before 2000 [30]. CRF01_AE (49.68%), CRF07_BC (22.26%), and CRF08_BC (21.93%) were the major strains circulating in 2006 [31]. CRF01_AE (43.2%), CRF07_BC (26.3%), CRF55_01B (8.5%) and CRF08_BC (8.4%) became the predominant strains circulating in 2013 [32]. In 2018, the proportion of individuals infected with CRF07_BC increased, while the proportion of individuals infected with CRF01_AE declined gradually. CRF07_BC was first identified from IDUs in the early 1990s and has spread to MSM [33]. In this study, CRF07_BC was confirmed as the most dominant HIV-1 genotype across MSM (40.65%, Fig. 1B), and the proportion of CRF07_BC in MSM increased from 33.3% in 2006[31] to 34.2% in 2013[32]. The CRF07_BC-infected cases are likely to keep increasing if HIV infection among MSM continue rapidly. Our finding highlights the important of CRF07_BC for HIV control in Guangdong.

The overall prevalence of TDR is 2.20% in Guangdong. In general, this prevalence has remained low according to WHO categorisation methods [34], and is lower than that in other regions of China [12–16]. A significant difference between the prevalence of TDR and CD4^+^ T cell count and genotype was observed, consistent with previous results [13]. When the CD4^+^ T cell count was used as a categorisation parameter, it was determined that patients with a CD4^+^ T cell count above 500 cells/mm^3^ were most likely to develop drug resistance. Of the six main genotypes, CRF07_BC had the lowest prevalence of TDR. In this study, TDR to NNRTIs and NRTIs was more common than TDR to PIs. This may be because NRTIs and NNRTIs are frequently used as first-line treatments. As the existence of TDR will affect antiretroviral therapy and spread drug resistance mutations, TDR continue to be monitored.

The SDRMs examined in our study were different from those in other regions. The most frequent PI-associated mutation in our study was M46L, whereas it is Q56E in southwest China [13], M46I in Iceland [35], and L90M in the south-central United States [36]. The most frequent NRTI-associated mutations in our study were M184V and L210W, while they are M41L and D67G in Southwest China [13] and T215C/D in Iceland and the south-central United States [35, 36]. The most frequent NNRTI-associated SDRM in our study was K103N, while it is V179E and V106I in Southwest China [13] and K103N/S and E138A in Iceland and the south-central United States [35, 36]. These dominant SDRMs are consistent with the main drug resistance sites among ART-treated patients in Guangdong [37]. The different SDRMs among different regions may be due to different genotype distributions or ART regimens.

To elucidate the transmission dynamics in the surveilled population, we constructed transmission clusters based on HIV-1 sequences. Of all the transmission networks, 53.09% included sequences from MSM. Moreover, more than half of the largest cluster, cluster A, and the second largest cluster, cluster B were comprised of sequences from MSM (68.36% and 54.84%, respectively). These results indicate that MSM may contribute significantly to the spread of the virus, and additional efforts should focus on this population for HIV prevention and control. Additionally, 28.85% (15/52) of patients infected by TDR strains were included in 9 clusters. A cluster (cluster C) containing HIV strains sharing the same SDRM (K103N) was found in the present study. The presence of TDR strains within transmission networks accounted for 4.64% (9/194) of all networks. These results indicate that HIV TDR may have spread in the transmission network, and the surveillance of TDR should be factored into treatment and prevention policies. Logistic regression analysis revealed that a CD4^+^ T cell count between 200 and 500 cells/mm^3^, the CRF07_BC strain and the CRF55_01B strain may be associated with the probability of entering the transmission network. The reasons for the association should be investigated further.

## Conclusions

In summary, this study of 2368 treatment-naïve HIV-1 patients shows that there is high genetic heterogeneity in Guangdong China. Although the overall prevalence of TDR is low, it is still necessary to remain vigilant to some important SDRMs.

## Data Availability

The datasets used and/or analyzed during the current study are available from the corresponding author on reasonable request.

## Abbreviations

a*ORs*: Adjusted odds ratios
aLRT: Approximate likelihood ratio test
ART: Antiretroviral therapy
COMET: Context-based Modeling for Expeditious Typing
CPR: Calibrated Population Resistance
CRF: Circulating recombinant form
EDTA: Ethylene diamine tetraacetic acid
HET: Heterosexual
IDU: Intravenous drug use
MSM: Men who have sex with men
NNRTI: Non-nucleoside reverse transcriptase inhibitor
NRTI: Nucleoside reverse transcriptase inhibitor
*ORs*: Odds ratios
PI: Protease inhibitor
SDRMs: Surveillance drug resistance mutations
TDR: Transmitted drug resistance
TN93: Tamura-Nei 93
95% *CIs*: 95% confidence intervals
